# Likelihood of identifying autistic traits with the autism spectrum quotient (AQ) in male juveniles with autism spectrum disorder (ASD) and severe behavioral problems (SBPs)

**DOI:** 10.1186/s12888-023-05200-1

**Published:** 2023-09-25

**Authors:** Alexa X. Rutten, Robert R. J. M. Vermeiren, Ilja L. Bongers, Chijs Van Nieuwenhuizen

**Affiliations:** 1 Centre for Child and Adolescent Psychiatry, GGzE, P.O. Box 909, Eindhoven, DP 8001, 5600 AX The Netherlands; 2 Scientific Center for Care and Wellbeing, Tilburg University, Tranzo, Tilburg, The Netherlands; 3 Department of Child and Adolescent Psychiatry, LUMC-Curium, Leiden University Medical Center, Leiden, The Netherlands; 4 Youz, Parnassia Group, The Hague, The Netherlands

**Keywords:** Screening ASD, AQ, Conduct disorder, Delinquency

## Abstract

**Background:**

When screening for autism spectrum disorders (ASD), the Autism Spectrum Quotient (AQ) is generally considered to be useful. Whether the AQ is also a suitable screener for ASD in juveniles with severe behavioral problems (SBPs) is unknown. Due to the overlap of symptoms between ASD and SBPs, particularly in juveniles low on empathy, the screening capacity of the AQ might be constrained. The aim of the present study was to investigate whether (comorbid) SBPs affect the screening capacity of the AQ. The hypothesis is that male juveniles with SBPs - but without a diagnosis of ASD - will score higher than male juveniles without both SBPs and ASD.

**Method:**

The AQ was completed by 216 male juveniles aged 15–18 years treated at an outpatient department of child and adolescent psychiatry. The 216 participants were categorized into four groups according to a clinical diagnosis of ASD and SBPs (defined as disruptive behavior disorder and/or delinquent behavior). Using multinomial logistic regression, we investigated whether the four identified groups, based on a diagnosis of ASD and SBPs, scored differently for the total score and subscales of the AQ.

**Results:**

Participants in the group with ASD (ASD^+^) but without SBPs (SBP^-^) were more likely to report higher levels of autistic traits than the reference group without both ASD and SBPs (ASD^-^SBP^-^), except for the subscale on attention to detail (ASD^+^SBP^-^ OR = 1.04; 95%CI = 0.98–1.11). Participants in the group with both ASD and SBPs were more likely to report higher levels for the total AQ score (ASD^+^SBP^+^ OR = 1.03; 95%CI = 1.00–1.05) and the communication subscale of the AQ (ASD^+^SBP^+^ OR = 1.18; 95%CI = 1.07–1.31) than the reference group without both ASD and SBPs.

**Conclusion:**

In outpatient male juveniles, SBPs do not affect the screening capacity of the AQ for autistic traits. In spite of the well-known overlap of symptoms between ASD and SBPs, male juveniles with SBPs but without a diagnosis of ASD do not score higher on the AQ than male juveniles without SBPs and without a diagnosis of ASD.

## Introduction

Autism Spectrum Disorder (ASD) is a neurodevelopmental disorder with social-communication deficits and restricted and repetitive behavior. Approximately 1 in 100 children are diagnosed with autism spectrum disorder around the world [1]. The importance of correct screening for autism spectrum disorder (ASD) is ubiquitous. Good diagnostic evaluation of ASD includes the use of instruments that are designed to assess multiple domains of functioning and behavior [2]. In 2014, the state-of-the-art literature in Europe concerning screening for ASD in young children was reviewed [3]. In this study, the importance of timely detection of ASD was emphasized. However, it was noted that screening outcomes should be interpreted with caution because they can be influenced by several factors, such as prevalence rates, age, level of functioning and autism severity, selection and formulation of items, cut-off criteria, informants and setting characteristics. However, whether the presence of severe behavioral problems (SBPs) is also a factor to be taking into account remains unknown.

The Autism Spectrum Quotient (AQ) was developed by Baron-Cohen and colleagues as a screener for ASD [4]. The AQ assesses five different areas: social skill, attention switching, communication, imagination and attention to detail. In 2006, the AQ was adapted for adolescents [5]. The AQ can be used to detect whether a person is suspected of having ASD. The findings of a study in a large general population, student sample and three matched patient groups indicate that the AQ, with 50 items within five domains, is a valuable instrument to assess where someone is on the autism spectrum [6]. However, in a study concerning the predictive validity of self-report questionnaires in the assessment of autism spectrum disorders in adults, was concluded that the predictive validity of two short versions of the AQ, the AQ-28 and the AQ-10, is not high enough to reliably predict a ASD diagnosis in outpatient settings [7]. In our study we used the 50-items-version of the AQ.

The AQ is also suitable for different age groups [8] and discriminating ASD traits from traits of other psychiatric disorders. For instance, the AQ differentiates significantly between ASD and attention deficit hyperactivity disorder at the group level [9]. However, there is also criticism pertaining to the AQ. In a study by Ashwood et al. [10], investigating adults suspected with ASD, self-report AQ scores did not significantly predict a clinical diagnosis of ASD. Furthermore, among adults with and without autism, eight items showed differences in response tendencies between adults with and without autism. The authors concluded that these differences were caused by items that were in general phrased negatively and that especially persons with autism have difficulties answering these items [11].

Severe behavioral problems in this study are defined as disruptive behavior disorder and/or delinquent behavior. Disruptive behavior disorders can be classified into oppositional defiant disorder and conduct disorder. Juveniles with disruptive behavior disorders have little empathy and concern for the feelings and wellbeing of others [12]. The median 12-month prevalence rate of disruptive behavior disorders is 6% with a range from 5 to 14% [13]. Juvenile delinquency in this study varies from property offences to violent offences. There is a tendence of declining (juvenile) delinquency on a global scale [14, 15].

The screening capacity of the AQ in juveniles with ASD and comorbid disruptive behavior disorders is still unknown. SBPs, including disruptive behavior disorders and delinquency, might affect the screening capacity of the AQ because of partial overlap with the manifestation of symptoms, particularly in relation to empathy deficits. Empathy deficits can be characteristic of both ASD and SBPs. In a study of boys aged 9–16 years, Jones and colleagues concluded that boys with psychopathic tendencies have difficulty reasoning with other people’s distress whereas boys with ASD have difficulty in knowing what other people think [16]. In the general population, high rates of callous-unemotional traits are usually strongly associated with SBPs, whereas adolescents with ASD show equally high rates of callous-unemotional traits but without SBPs, and probably having similar affective deficits [17].

Therefore, the aim of this study is to investigate whether (comorbid) SBPs affect the screening capacity of the AQ. The hypothesis is that male juveniles with SBPs but without a diagnosis of ASD will score higher on a number of subscales of the AQ than male juveniles without both severe behavioral problems and without ASD. To this end, male juveniles with and without ASD and with and without SBPs who were treated at an outpatient department of child and adolescent psychiatry were investigated. Four groups of male juveniles were distinguished: with ASD but without SBPs (ASD^+^SBP^−^); with both ASD and SBPs (ASD^+^SBP^+^); without both ASD and SBPs (ASD^−^SBP^−^); and without ASD but with SBPs (ASD^−^SBP^+^).

## Methods

### Participants

All participants were male juveniles who were treated at an outpatient department of child and adolescent psychiatry in The Netherlands. The participants were 15 up to and including 18 years old. Specific data on socioeconomic status and race or ethnicity were not recorded. This study was part of a more comprehensive study focusing on ASD in male juveniles with severe behavioral problems, therefore only males were included in the present study.

**Fig. 1:**
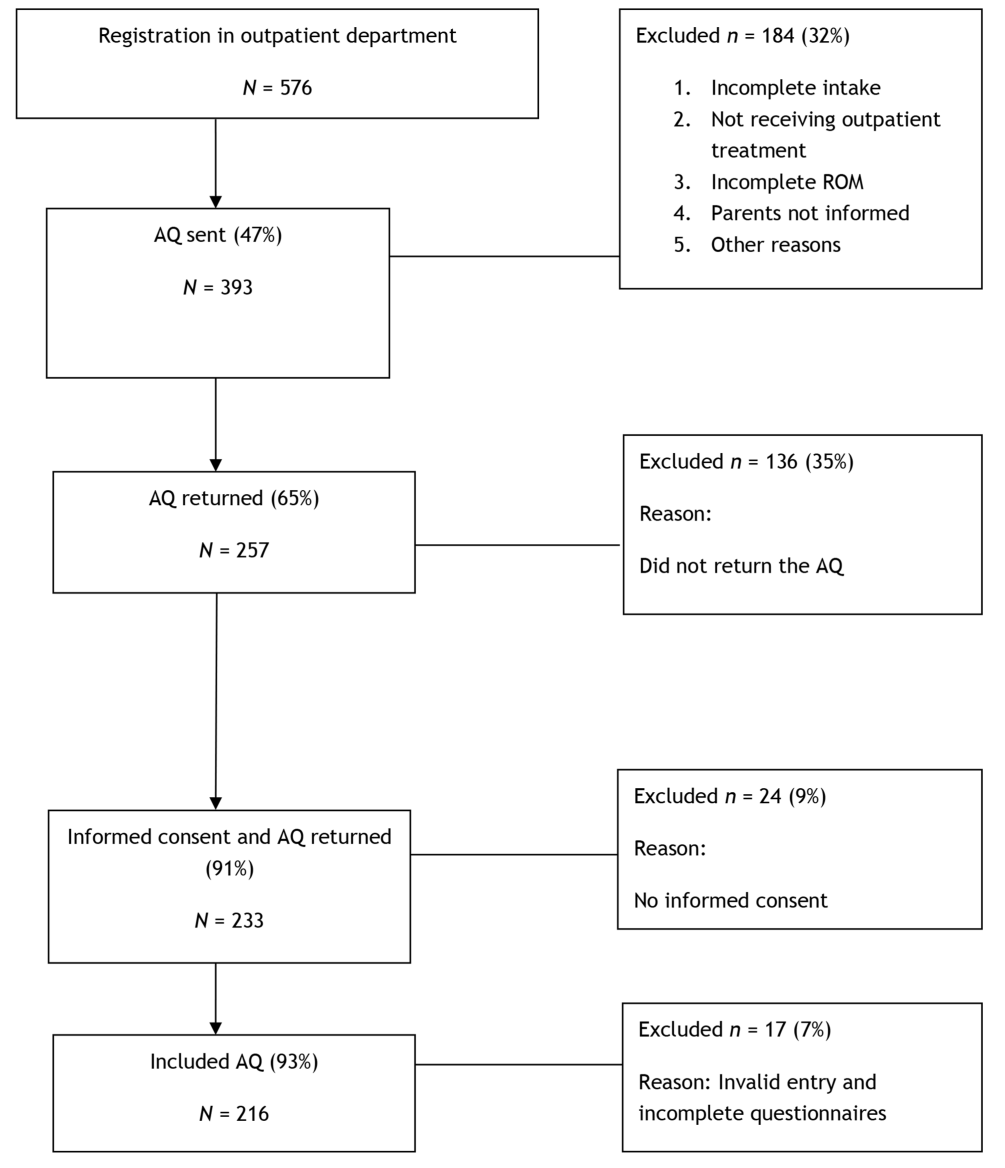
Flowchart of excluded patients. AQ = Autism Spectrum Quotient; ROM = routine outcome monitoring

As shown in Fig. 1, the initial sample comprised 576 participants. A total of 184 participants were excluded from the sample for the following reasons: the intake session was not completed (*n* = 100); they were not receiving outpatient treatment (*n* = 63); they did not complete the routine outcome monitoring (*n* = 4); they did not receive the questionnaires because their parents were not informed (*n* = 2); or other reasons (*n* = 15). A total of 257 participants returned the AQ questionnaire, from which 24 participants were excluded because there was no informed consent and a further 17 participants were excluded because of invalid entry or an incomplete questionnaire. This resulted in a final sample of 216 participants. Community members were not involved in the study.

### Measurements

#### Autism spectrum quotient (AQ)

The AQ is a continuous, quantitative self-report measure of autistic traits. The 50 items of the AQ are classified into five subscales: social skill, attention switching, communication, imagination and attention to detail. All items are scored on a four-point scale (with response options of ‘agree’, ‘slightly agree’, ‘slightly disagree’ and ‘disagree’). The Baron-Cohen method of scoring was used, where the two highest and two lowest scores for an item are combined in a dichotomous response [4]. A cut-off point of 30 for the total AQ score is recommended in an article using the adolescent version of the AQ [5]. The authors proposed that a cut-off of 30 might be considered in future screening studies with adolescents because the percentage of those with autism (especially those with Asperger’s syndrome) scoring above a cut-off point of 32 is much lower. We thus maintained a cut-off point of 30. The internal consistency of items in each of the five subscales was calculated and Cronbach’s alpha coefficients were all considered as moderate (0.50–0.70) to sufficient (≥ 0.70): social skill, 0.77; attention switching, 0.67; communication, 0.6; imagination, 0.65; and attention to detail, 0.63 [4].

#### Structured file analysis

Structured file analysis was used to register DSM-IV-TR ASD classifications and SBPs (namely disruptive behavior disorders and delinquent behavior). The DSM-IV-TR diagnoses were based on clinical evaluation by a (child and adolescent) psychiatrist, a healthcare psychologist or a clinical psychologist. All juveniles were widely screened for psychiatric problems, not only focused on the registered complaint.

SBPs were defined as conduct disorders, oppositional defiant disorders and/or delinquent behavior. The delinquent behavior varied and included traffic violation and order disturbance, drug offence, vandalism, property offence without violence, moderate violent offence, violent property offence, serious violent offence and sex offence. Although truancy is a criminal offence under Dutch law, this was excluded because it is usually not an expression of (a pattern of) delinquent behavior.

Based on the clinical evaluation and the SBPs, the following four distinct groups of male juveniles were created: with ASD but without SBPs (ASD^+^SBP^-^); with both ASD and SBPs (ASD^+^SBP^+^); without both ASD and SBPs (ASD^-^SBP^-^); and without ASD but with SBPs (ASD^-^SBP^+^). In the analyses, these four groups are used.

### Procedure

The participants completed the AQ in their own time, on their own, in the home-situation, shortly after admission to the outpatient department of child and adolescent psychiatry. After completing the questionnaire they sent it back to the principal researcher (A.X.R.). Analysis of the AQ file was performed by the principal researcher (A.X.R.) and a trainee. All files were completed by means of consensus scoring until an interrater reliability of at least 80% was achieved. Prior to the start of the study, the Medical Ethics Committee of St. Elisabeth Hospital Tilburg approved the study (METC No. NL41160.008.12/P1257).

### Statistical analyses

An attrition analysis was performed to investigate the generalizability of the sample. The included and excluded male juveniles were compared on specific background characteristics. Age and AQ total score did not differ significantly (F(1,217) = 2.39, p = 0.12; F(1,255) = 3.62, p = 0.06, respectively), also DSM classifications did not differ (range chi-square = 0.01–2.21, range p = 0.99–0.19). The t-tests were used for continuous variables whereas chi-square tests were used for categorical variables. Using multinomial logistic regression, we investigated whether the four identified groups, based on ASD and SBPs, scored differently for the total score and subscales of the AQ. Odds ratios were estimated for each subscale of the AQ separately in order to determine the univariate associations between the four groups and the AQ. In all analyses, the ASD^-^SBP^-^ group was the reference category. All analyses were performed using SPSS and effects were considered significant if *p* < 0.05.

## Results

### Background characteristics

Participants were males with a mean age of 16.3 years (SD = 1.1, range = 14–18). In the total group (N = 216 male juveniles), according to the information in the electronic patient file, 113 (52.3%) participants were classified with a diagnosis of ASD and 51 (23.6%) participants with SBPs; see Table 1 for the number of participants in the four groups based on the clinical evaluation and level of SBPs. The distribution of SBPs was the same among the participants with and without ASD [χ^2^(1) = 0.377; *p* = 0.539].

**Table 1:** Number of male juveniles in the four groups with a clinical diagnosis of ASD and/or SBPs

	SBP^−^	SBP^+^		Total
ASD^+^	88	25		113
ASD^−^	77	26		103
Total	165	51		216

*Note.* ASD^+^ = with autism spectrum disorder; ASD^−^ = without autism spectrum disorder; SBP^+^ = with severe behavioral problems; SBP^−^ = without severe behavior problems;

Table 2 shows the means for the total score and subscales of the AQ. In all groups, the scores were below the cut-off as defined by Baron-Cohen et al. [4]. The total AQ score was 21.17 for the ASD^-^SBP^-^ group, 20.48 for the ASD^-^SBP^+^ group, 23.46 for the ASD^+^SBP^-^ group and 22.74 for the ASD^+^SBP^+^ group. The means differed significantly [*F*(3,212) = 7.90; *p* = 0.000] and the post hoc test indicated that the mean for the ASD^+^SBP^-^ group was significantly larger than the means for the ASD^-^SBP^-^ and ASD^–^SBP^+^ groups.

**Table 2:** Mean (SD) AQ scores for the four groups

	ASD^−^SBP^−^	ASD^−^SBP^+^	ASD^+^SBP^−^	ASD^+^SBP^+^	*F*-test	Post hoc
Total AQ score	21.17 (3.22)	20.48 (3.00)	23.46 (3.89)	22.74 (4.12)	7.90*	ASD^+^SBP^−^ > ASD^−^SBP^−^, ASD^–^SBP^+^
Social skill	19.32 (5.45)	17.62 (5.13)	22.82 (5.82)	20.76 (6.03)	8.27*	ASD^+^SBP^−^ > ASD^−^SBP^−^, ASD^–^SBP^+^
Attention switching	23.55 (5.19)	22.35 (5.75)	25.85 (6.25)	24.08 (4.96)	3.64*	ASD^+^SBP^−^ > ASD^–^SBP^+^
Communication	19.96 (4.54)	20.15 (4.35)	22.74 (4.85)	23.72 (5.47)	7.17*	ASD^+^SBP^−^ > ASD^−^SBP^−^ ASD^+^SBP^+^ > ASD^−^SBP^−^, ASD^–^SBP^+^
Imagination	21.83 (3.88)	21.50 (3.78)	23.69 (4.78)	23.72 (5.15)	3.55*	ASD^+^SBP^−^ > ASD^−^SBP^−^
Attention to detail	21.18 (4.29)	20.81 (4.84)	22.22 (5.60)	21.40 (5.67)	0.83	

*Note.* AQ = Autism Spectrum Quotient; ASD^+^ = with autism spectrum disorder; ASD^−^ = without autism spectrum disorder; SBP^+^ = with severe behavioral problems; SBP^−^ = without severe behavioral problems; * *p* < 0.05

The multinomial logistic regression results for whether SBPs affect the screening capacity of the AQ are depicted in Table 3. Participants in the ASD^+^SBP^-^ group were more likely to report higher levels of autistic traits than the reference ASD^-^SBP^-^ group, except for the subscale of attention to detail (ASD^+^SBP^-^ Nagelkerke R^2^ = 0.01; OR = 1.04; 95%CI = 0.980–1.11). Participants in the ASD^+^SBP^+^ group were more likely to report higher levels for the total AQ score (Nagelkerke R^2^ = 0.11; OR = 1.03; 95%CI = 1.00–1.05) and the subscale of communication (Nagelkerke R^2^ = 0.10; OR = 1.18; 95%CI = 1.07–1.31) compared to the reference ASD^-^SBP^-^ group. The ASD^-^SBP^+^ group was not likely to report higher levels of autistic traits on any of the subscales compared with the reference group.

**Table 3:** Results of multinomial logistic regression for the total score and subscales of the AQ (OR, 95%CI) in the four groups

	Total AQ score	Social skill	Attention switching	Communication	Imagination	Attention to detail
Chi -square	23.23*	24.34*	10.87*	20.88*	10.62*	2.50
Nagelkerke *R*^2^	0.11	0.12	0.05	0.10	0.05	0.01
ASD^−^SBP^+^	0.99 (0.96–1.02)	0.93 (0.84–1.02)	0.96 (0.88–1.04)	1.01 (0.92–1.11)	0.98 (0.88–1.09)	0.98 (0.90–1.08)
ASD^+^SBP^−^	1.04 (1.02–1.06) *	1.114 (1.05–1.18) *	1.07 (1.02–1.14) *	1.13 (1.06–1.22) *	1.10 (1.03–1.19) *	1.04 (0.98–1.11)
ASD^+^SBP^+^	1.03 (1.00–1.05) *	1.05 (0.97–1.14)	1.01 (0.94–1.10)	1.18 (1.07–1.31) *	1.10 (1.00–1.22)	1.01 (0.92–1.11)

*Note.* The reference group for multinomial logistic regression was ASD^−^SBP^−^ in all analyses; AQ = Autism Spectrum Quotient; ASD^+^ = with autism spectrum disorder; ASD^−^ = without autism spectrum disorder; SBP^+^ = with severe behavioral problems; SBP^−^ = without severe behavioral problems; * *p* < 0.05

## Discussion

The aim of the present study was to investigate whether (comorbid) SBPs affect the screening capacity of the AQ. The current study therefore examined the AQ scores in an outpatient sample of male juveniles with and without ASD and with and without comorbid SBPs. We hypothesized that male juveniles with SBPs but without a diagnosis of ASD would score higher on a number of subscales of the AQ than male juveniles without SBPs. However, the juveniles with SBPs but without ASD did not report increased levels on the AQ when compared to the juveniles without SBPs and without ASD, indicating that SBPs do not affect the screening capacity for autistic traits. Consequently, the total AQ score distinguished male juveniles with ASD from male juveniles without ASD.

We expected that especially problems in empathy and interaction with other peers cause the commonalities between SBPs and ASD. In contrast to what we expected, the hypothesis cannot be confirmed that male juveniles without ASD but with SBPs will score higher on the AQ than those without both ASD and SBPs. With the well-known overlap of symptoms between SBPs and ASD, we assumed that it was more likely that juveniles with SBPs would report problems in empathy and social skills compared to juveniles with ASD. In a study on cognitive and affective empathy in children with conduct problems, Pasalich et al. found evidence that children with high levels of callous-unemotional traits and ASD symptoms may have the most pronounced deficits in affective empathy [18]. Our study nonetheless demonstrates that in juveniles with ASD and comorbid SBPs the AQ score is higher than in juveniles without ASD and with SBPs. One possible reason why the hypothesis could not be confirmed is that only a few items of the AQ pertain to empathy. In summary, the total AQ score differentiates male juveniles with ASD from male juveniles without ASD, even in the case of comorbid behavioral problems.

Individuals with ASD have the tendency to score lower on self-report ASD questionnaires such as the AQ [19]. This tendency can be explained by the ASD-related typical restricted self-awareness [19] or the more self-reported empathic features [20]. In our study, there was also a tendency to score lower on the AQ in all four groups. However, this tendency was not associated with specific ASD-related features. In our study, all male juveniles scored below the designated screening cut-off. Despite those low scores, the total AQ score distinguishes male juveniles with ASD from male juveniles without ASD, as classified by clinical diagnosis, even in the group with comorbid SBPs. Therefore, the screening capacity of the AQ is not affected by the SBPs.

### Study strengths and limitations

The strength of this study is the number of male juveniles who completed the AQ. Four groups of male juveniles could be formed: groups with and without ASD and with and without comorbid SBPs. A limitation of this study was that we only included male juveniles treated at an outpatient department of child and adolescent psychiatry. It is possible that male juveniles with more SBPs are less inclined to be referred to and treated at an outpatient department of child and adolescent psychiatry and consequently were not included in this study. It can be considered as a limitation to exclude females from the study. This study was, however, part of a more comprehensive study focusing on ASD in male juveniles with severe behavioral problems in which only males were included. Females with ASD can be under-diagnosed because of differences in symptomatology [21–23]. In future research in this area, it is important to include females and compare results between males and females in manifestation of symptoms of ASD and SBPs. However, since there are no indications that those differences in symptomatology do concern the overlapping symptomatology of ASD and SBPs, we expect therefore that it is possible to generalize the findings of this study to female juveniles with SBPs. Future research might uncover whether the results in another study population, such as in a youth custodial institution, differ because of the possibility of more SBPs. Another suggestion for future research is to revise the wording of the AQ to a more contemporary language that will be more appropriate to the current world of juveniles.

## Conclusion

In spite of the well-known overlap of symptoms between ASD and SBPs, in an outpatient population, male juveniles without ASD but with SBPs do not score higher on the AQ than those without both ASD and SBPs. These results suggest that the AQ can be used to identify autistic traits, even among male juveniles with SBPs. Moreover, it is important to keep in mind that the AQ is not a diagnostic instrument but a screener to identify persons with autistic traits. For a classification of ASD, further psychiatric examination is always necessary.

## Data Availability

The datasets used and/or analyzed during the current study are available from the corresponding author upon reasonable request. The datasets analyzed during the current study are not publicly available due to intellectual property rights. The research group conducted quality checks on the data during the project to check that they were complete, correct and consistent.

## List of abbreviations

ASD: Autism spectrum disorder
AQ: Autism Spectrum Quotient
SBPs: Severe behavior problems
